# From across the Seas

**Published:** 1919-10-11

**Authors:** 


### FROM ACROSS THE SEAS.

### News from New Zealand.

The Journal issued by the Department of Public Health of New South Wales is now half-way through the second volume, and it is claimed that the interest expressed in it by hospital workers and charitable organisations is increasing. The latest number has useful articles of a medical and administrative nature, including one in respect of the recent Diphtheria Epidemic, and another dealing with the Economic Administration of the Epidemic Expenditure. It is the present policy of the Public Health Department to encourage voluntary contributions towards the maintenance of hospitals, and a subsidy at the rate of 24s. to each £1 is paid on all ordinary donations which are given to a hospital unconditionally.

The Hospital and Charitable Aid Board evidently have to do with charitable relief outside hospitals, as a case recently arose where the children of a family, whose father had been hanged by the State for the murder of the mother, had become dependent upon a Hospital Board for maintenance, and it was ruled that as the law stands at present it is the duty of the local Hospital Board to provide relief for the members of such a family if in necessitous circumstances.

